# Intelligent Industrial Cleaning: A Multi-Sensor Approach Utilising Machine Learning-Based Regression

**DOI:** 10.3390/s20133642

**Published:** 2020-06-29

**Authors:** Alessandro Simeone, Elliot Woolley, Josep Escrig, Nicholas James Watson

**Affiliations:** 1 Intelligent Manufacturing Key Laboratory of Ministry of Education, Shantou University, Shantou 515063, China; simeone@stu.edu.cn; 2 Wolfson School of Mechanical, Electrical and Manufacturing Engineering, Loughborough University, Loughborough LE11 3TU, UK; E.B.Woolley@lboro.ac.uk; 3 i2CAT Foundation, Calle Gran Capita, 2 -4 Edifici Nexus (Campus Nord Upc), 08034 Barcelona, Spain; josep.escrig@i2cat.net; 4 Food, Water, Waste, Research Group, Faculty of Engineering, University of Nottingham, University Park, Nottingham NG7 2RD, UK

**Keywords:** ultrasonic sensors, optical sensors, machine learning, regression, artificial neural networks, Clean-in-Place, digital manufacturing, industry 4.0, process optimisation

## Abstract

Effectively cleaning equipment is essential for the safe production of food but requires a significant amount of time and resources such as water, energy, and chemicals. To optimize the cleaning of food production equipment, there is the need for innovative technologies to monitor the removal of fouling from equipment surfaces. In this work, optical and ultrasonic sensors are used to monitor the fouling removal of food materials with different physicochemical properties from a benchtop rig. Tailored signal and image processing procedures are developed to monitor the cleaning process, and a neural network regression model is developed to predict the amount of fouling remaining on the surface. The results show that the three dissimilar food fouling materials investigated were removed from the test section via different cleaning mechanisms, and the neural network models were able to predict the area and volume of fouling present during cleaning with accuracies as high as 98% and 97%, respectively. This work demonstrates that sensors and machine learning methods can be effectively combined to monitor cleaning processes.

## 1. Introduction

The cleaning of surface fouling is important in many industries to ensure equipment, such as heat exchangers, are operating under optimal conditions. Cleaning is even more important within food and drink manufacturing, as it ensures that equipment is hygienic and eliminates the cross-contamination of ingredients between production batches. The cleaning of equipment was historically performed manually by factory operators who would be required to disassemble equipment and spray internal surfaces with water and cleaning chemicals. Currently, most of the industrial cleaning of equipment is performed by automated systems named Clean-in-Place (CIP). These systems feature process tanks for cleaning fluids and pumps, pipework, and spray balls to perform the cleaning within processing equipment, without the need for disassembly. Clean-in-Place systems clean the equipment by a combination of mechanical flow, chemicals, temperature, and time, and they generally feature several steps including initial rinse, detergent wash, post detergent rinse, and sterilant [1].

Although a significant improvement over manual cleaning, CIP comes at a cost. The economic cost of CIP is primarily associated with lost production time. Every minute spent cleaning equipment is time that the equipment cannot be used to manufacture product. As the food and drink manufacturing sector operates on tight profit margins, reducing production downtime is extremely desirable. In addition, there are financial costs associated with the use of cleaning chemicals, water, and energy use. The main environmental cost of cleaning is the vast amount of water and energy utilized. Statistics show that cleaning accounts for 30% of the energy used in dairy production [2] and 35% of the water used in beer brewing [3]. Due to the costs associated with CIP, research has been performed to optimize the process. The vast majority of this work has focused on either studying the cleaning settings, such as fluid temperature and flow rate [4] or studying the actual mechanisms of surface fouling and cleaning [5].

### 1.1. Sensor Research in Cleaning

Wide literature and industrial practice are available on industrial cleaning processes monitoring using different sensor technologies. Most research efforts are directed on either: (1) monitoring the cleaning fluid properties to determine if it contains soiling material or cleaning fluids, or (2) monitoring the internal surfaces of the processing equipment. Monitoring the properties of fluid in the systems has been performed using electrical and optical methods [6,7,8]. These techniques offer valuable insight but are not sensitive to fouling that is still adhered to a surface so can often produce misleading results on how clean the equipment actually is. 

A variety of different sensor methods have been utilized to monitor the presence of surface fouling with the main ones being electrical (e.g., [9,10,11,12]), optical (e.g., [13,14,15,16,17]), acoustic (e.g., [13,18,19]) and ultrasonic methods (e.g., [20,21,22,23]). Electrical methods utilize sets of probes and measure electrical properties such as resistance and conductance across pairs of probes. The presence of fouling affects heat transfer in pipes, which is detected by the electrical methods. Although a promising technique, the recorded signals can become noisy and difficult to interpret when turbulent flow is used, which is nearly always the case in CIP. Optical method utilizing either florescence [13] or fibre-optic [17] techniques have been used to monitor surface fouling removal. Optical techniques can often provide spatial information of the location and degree of fouling; however, lighting is required, which is extremely challenging in pipe work. Acoustic methods use mechanical instruments to excite a vibration in the equipment where the fouling is present. These vibrations are subsequently detected by a transducer (generally piezoelectric). Acoustic methods have been used to monitor the removal of materials, including dairy fouling [24] and shampoo residue [18] from a surface. In general, the presence of fouling will damp the vibrations detected by the transducers. However, it will be challenging to deploy these methods in industrial environments where other vibrations exist. 

Ultrasonic (US) sensor technologies employ high-frequency mechanical waves, which propagate in the system under inspection. The benefits of US sensors is that they are often significantly cheaper than other techniques and can perform non-destructive measurements in real time for a variety of different industrial applications [19]. The application of US techniques to monitor fouling was pioneered by Withers [13], who used two US transducers in a laboratory scale rig filled with a fluid and different measuring the thickness of diverse food and non-food fouling materials on the internal surfaces [13]. Since then, research activities have been carried out utilizing US technologies for fouling detection and cleaning monitoring [19,21,22,23,25,26,27]. The majority of previous works employed single US sensors operating in reflection mode and has mainly focused on dairy fouling. The focus has often been on dairy fouling, as it is especially difficult to clean and a current challenge for the sector. 

These different sensor methods vary in terms of their cost, complexity, and operating parameters such as speed of data acquisition and spatial area monitored. It is clear that no single sensor method is suitable for monitoring all the different types of equipment used within food production, so a range of different sensor and data analysis methods should be deployed to monitor the different components in a CIP system. 

With any sensor technique, there is a need for appropriate data analysis methods to link the recorded measurement to the system under inspection. Machine learning is an attractive data analysis method as the development of first-principle models, which are challenging for real-life applications, is not required. Supervised machine learning methods enable the development of models with a labelled dataset and have been used successfully to monitor surface fouling removal during cleaning processes [20,21,22,23]. All of this previous work has developed classification models to determine when the surface remains fouled or is clean using ultrasonic measurements. This previous work has focused on dairy and other fouling materials. The majority of this work has been performed on flat test sections, but examples exist [20] of the technique being used in pipes of circular cross-sections.

### 1.2. Signal and Image Processing

Recorded US waves require appropriate signal processing to extract key information. Wavelet decomposition (WD) decomposes a single time domain signal instance (e.g., ultrasonic) into a two-dimensional function, where each of the decomposed signals is a mixture of source signals [28]. It can be considered as a series of band pass filters, whose results could be regarded as different mixtures of independent source signals [29]. Compared to Fast Fourier Transformation, WD provides time and frequency combined information for more efficient feature extraction from a computational effort perspective. For this reason, such algorithms are very advantageous in real-time data processing, resulting in one of the most widespread and powerful methods of signal analysis [30]. A variant of this method, the wavelet packet transform (WPT), which is a generalization of the wavelet decomposition [31], is used in this work to process the ultrasonic signals and extract features used in the neural networks. 

With reference to CIP processes, the literature reports a number of image segmentation procedures for food fouling detection. A review reported in [14] compares Otsu, Iteration method, 1D and 2D entropy. Fuzzy c-means (FCM) clustering [32,33] is a powerful clustering algorithm that allows each data point to belong to multiple clusters with varying degrees of membership. The main advantages are represented by its property of convergence and low complexity. Xiong et al. [34] utilized the FCM concept to perform image segmentation on RNAi Fluorescence Cellular Images setting the three-class concept to cluster the pixel values. The threshold is obtained by averaging the maximum in the class with the smallest centre and the minimum in the class with the middle centre. From an image histogram analysis perspective, the threshold results are to be located in correspondence of the demarcation line between the first and the second peak.

Intelligent decision-making on the cleaning state is enabled by machine learning paradigms; in this respect, a commonly utilized technique is the Neural Network (NN) due to the high-performance capabilities in nonlinear modelling using parallel processing [35] pattern recognition (classification), time series prediction, and data regression. 

As regards classification purposes, the literature provides a variety of applications in cleaning processes, such as [20], in which ultrasonic measurements are used as input to a NN to monitor the fouling removal of food materials in plastic and metal cylindrical pipes. Moreover, [23] utilized acoustic features and fed them together with temperature and mass flow rate (both measured) into a NN to make the decision of fouling presence or absence. 

A time series prediction-based intelligent decision-making support system is developed in [14] utilizing nonlinear autoregressive models with exogenous inputs (NARX) Neural Network was adopted and configured, trained, and tested to predict the cleaning time based on the image processing results.

Concerning the use of NN for regression purposes in cleaning processes, the available research is considerably limited, such as in applications to heat exchanger fouling assessment [36] during ultrasonic cleaning using Convolutional Neural Networks (CNN).

This research will monitor the cleaning of different food fouling materials in a bespoke benchtop rig using ultrasonic (US) and optical sensors. The US signals are analysed using wavelet methods, whilst novel image processing methods are developed to calculate the number of pixels in an image’s Region of Interest (ROI) that contain fouling and the total fouling volume. Results from both sensors are assessed to determine their capability in monitoring cleaning processes, and features extracted from the ultrasonic sensor measurements are used to develop a Neural Network regression model that can predict the amount of fouling present and be used to determine when the cleaning processes will be complete. This is the first time that US measurements have been used to develop regression ML models to predict the degree of fouling. It was decided to develop the regression models using the US measurements, as optical measurements would not be possible in processing equipment geometry such as closed pipework. 

## 2. Materials and Methods

This section introduces the experimental and computational methodologies utilized for the design and realization of the cleaning tests along with the signal and image processing procedures adopted to estimate the remaining fouling during the cleaning process.

### 2.1. Experimental Tests

**Figure 1:**
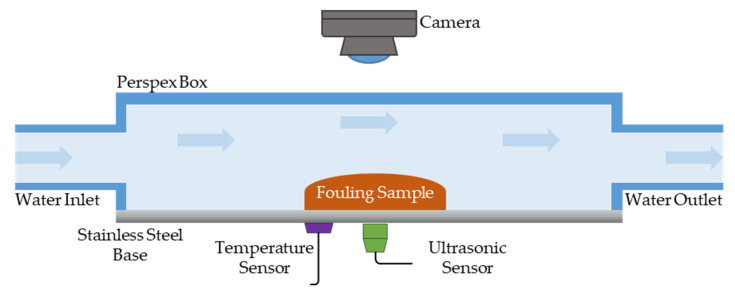
Experimental setup with sensors.

A benchtop experimental flow rig was constructed for the fouling removal experimental tests (Figure 1). The rig featured a 1.2 mm thickness stainless steel (SS 430) bottom plate where the fouling removal would be monitored. In order to allow for image acquisition during fouling removal during cleaning, the rig lateral and upper surfaces were made of transparent Perspex. The rig was 300 mm long with an internal width and height of 40 mm. Valves controlling mains water pressure were located at either end of the rig to allow fluids to flow through and perform the cleaning. 

The fouling materials selected for the experimental tests were tomato paste, gravy, and concentrated malt extract. This range of different materials was utilized to investigate the different fouling and cleaning mechanisms [37,38]. Full details of the fouling materials are available in [27].

The particular choice of the fouling materials, namely tomato paste, gravy, and concentrated malt was made as they represent common materials from the food and drink manufacturing sector. In addition, they all have a different composition and therefore foul and clean from a surface differently. It has been shown in [39] that different materials yield different cleaning characteristics, and it is important to assess the potential of the developed sensing techniques for a range of industrially relevant scenarios. Such considerations highlight the need for more adaptive CIP systems in order to guarantee the removal of all fouling whilst avoiding over-cleaning.

The preparation of the gravy fouling included mixing 10 grams of granules and 10 mL of tap water in a beaker at 70 °C for 1 min with continuous stirring. The other fouling media did not require preparation. The fouling layer was obtained by applying 15 grams of the material on to the centre of the bottom plate of the rig (in correspondence of the US transducer, which was attached on the opposite side of the plate). Then, the fouling was manually spread using a spatula to form an even layer of 5 mm thickness. The cleaning experimental tests were initiated by opening the inlet valve on the rig to let it slowly fill with water. Then, data acquisition was started, and the outlet valve was opened. In this way, water flowed through the rig whilst removing the fouling material. Each cleaning test continued until the fouling appeared to be completely removed, which was determined via the camera images and visual observation. This was to ensure that enough data was recorded for both a range of fouled and clean conditions. 

The experimental rig was endowed with a US sensor, a camera, and a temperature sensor to acquire data during the cleaning tests. The sensor’s placement within the experimental rig is shown in Figure 1. The US sensor was a 5 MHz magnetic contact transducer (Olympus^®^, Tokyo, Japan). This was mounted to the bottom side of the rig by using a thin film of couplant fluid between the US sensor and the SS430 wall to enable transmission of US waves into the material. A Bayonet Neill–Concelman (BNC) cable was used to connect the US transducer to a US box (Lecoeur Electronique^®^, Chuelles, France). The US box performed the role of exciting the US transducer with an electrical voltage flat and recording and digitize the received US waves. A Logitech^®^( Lausanne, Switzerland) C270 3MP web camera was employed to acquire digital images, while the temperature was recorded with a RTD PT100 connected to a Pico Technology^®^ (Cambridgeshire, UK) PT-104 data logger. The US box, PT-104 data logger, and camera were all connected to a laptop and controlled through specifically designed software developed in MATLAB^®^. During the cleaning experiments, US and temperature data were sampled at 0.25 Hz, while images were acquired every 20 s (0.05 Hz). 

By varying and combining the fouling material and the temperature, an experimental program was defined and reported in Table 1. Each test was repeated a number of times to increase the statistical reliability of the experimental campaign.

### 2.2. Data Processing and Features Extraction

The estimation of the fouling surface and volume is carried out via intelligent regression based on features extracted from ultrasonic signals and information computed via image processing. 

**Figure 2:**
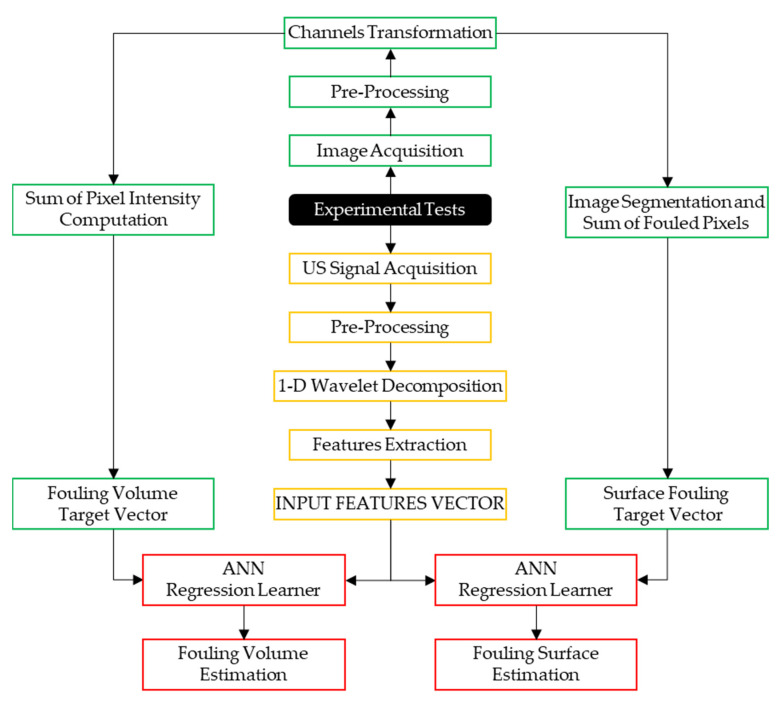
Data acquisition and processing flow chart.

The framework adopted for this paper is illustrated in Figure 2, and it is composed of three main parts: ultrasonic signal processing (yellow boxes), aimed at extracting significant information on the cleaning process; image processing (green boxes) aimed at computing the amount of fouling surface and volume in correspondence to the acquired images; and machine learning-based regression (red boxes) aimed at performing an estimation of surface and volume fouling during the cleaning process.

#### 2.2.1. Signal Processing

Due to the different sampling rate of the digital camera and the ultrasonic sensor, a pre-processing phase was carried out on each signal instance to allow for further processing.

**Figure 3:**
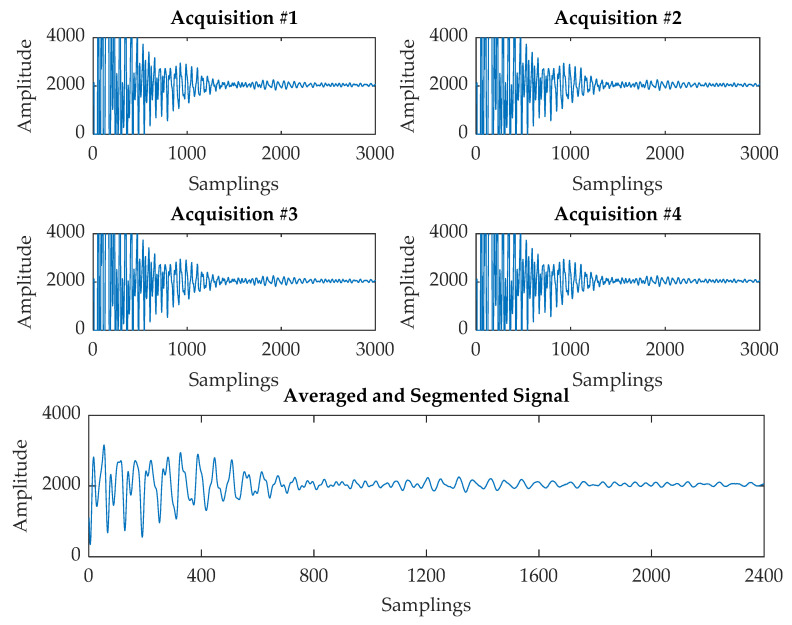
Ultrasonic (US) signal instance log.

Each raw US signal instance log (s) includes four acquisitions distributed over a time span of 37.5 μs, as shown in Figure 3. Therefore, the first pre-processing step consists of averaging the four signals and subsequently, in order to match the US signals and the image acquisition sampling rates, the four signal instances between each image acquisition log are averaged as per Equation (1), where n=4 indicates the four acquisitions and m=4 indicates the four signal instances recorded between each image acquisition.

(1) $$\bar{s}=\frac{1}{n}\frac{1}{m}\overset{n}{\underset{i=1}{\sum }}\overset{m}{\underset{j=1}{\sum }}s_{ij}$$

Then, such an averaged signal was subject to a segmentation procedure in order to remove the saturated signal portion corresponding to the first 6000 samplings. The result is a segmented signal made of 2400 samplings reported in Figure 3.

**Figure 4:**
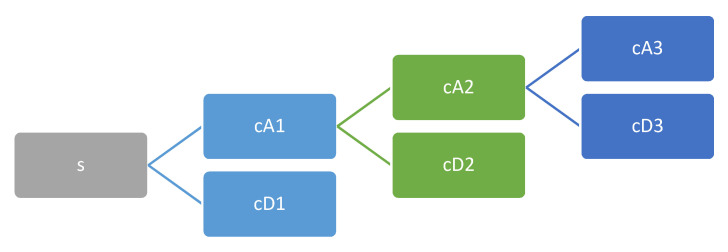
Wavelet decomposition tree.

The WPT [40] of a sensor signal generates packets of coefficients computed via scaling and shifting from a selected mother wavelet function. In this way, at the first level of WPT, the original sensor signal S is split into two frequency band packets, which are called approximation cA1 and detail cD1. Likewise, at the second level, each approximation and detail packet are again split into further approximations and details cA2 and cD2, respectively, and the process is repeated until the required level for the application is reached [41], generating a “tree” of decomposition packets, as shown in Figure 4.

**Figure 5:**
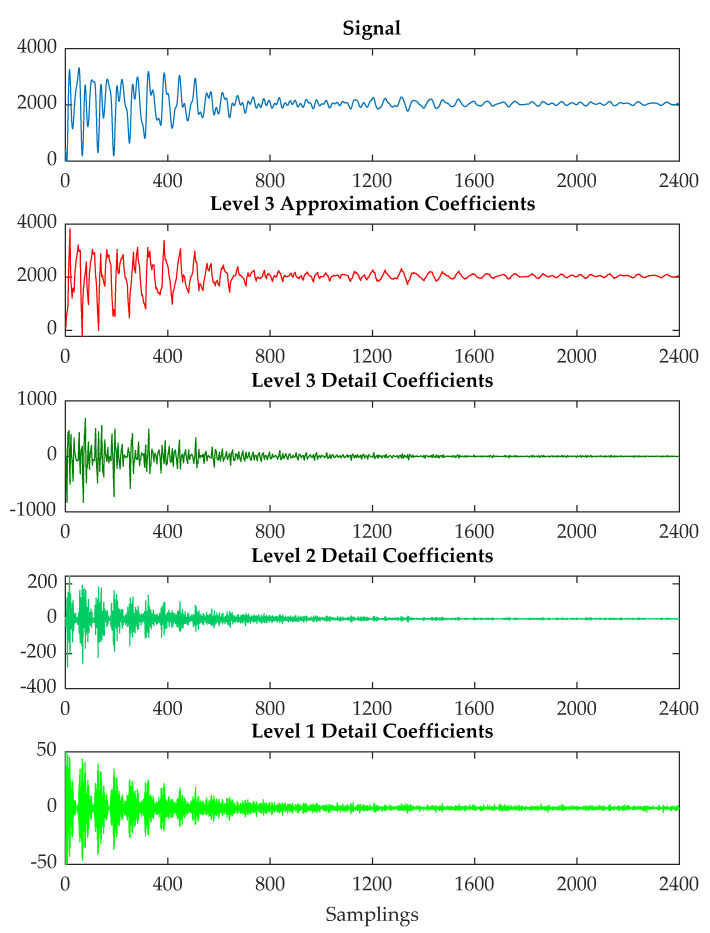
Level 3 full decomposition.

In this paper, the WPT is utilized to process the US signal instances with the aim of extracting significant features on the cleaning state over time. In this respect, a three-level wavelet decomposition was performed on the segmented signal using the order 3 Daubechies mother wavelet [42]. In this way, the coarse scale approximation coefficients and the detail coefficients from the decomposition were extracted from each US signal. Figure 5 displays an example of signal instance and its related details and approximation coefficients.

**Figure 6:**
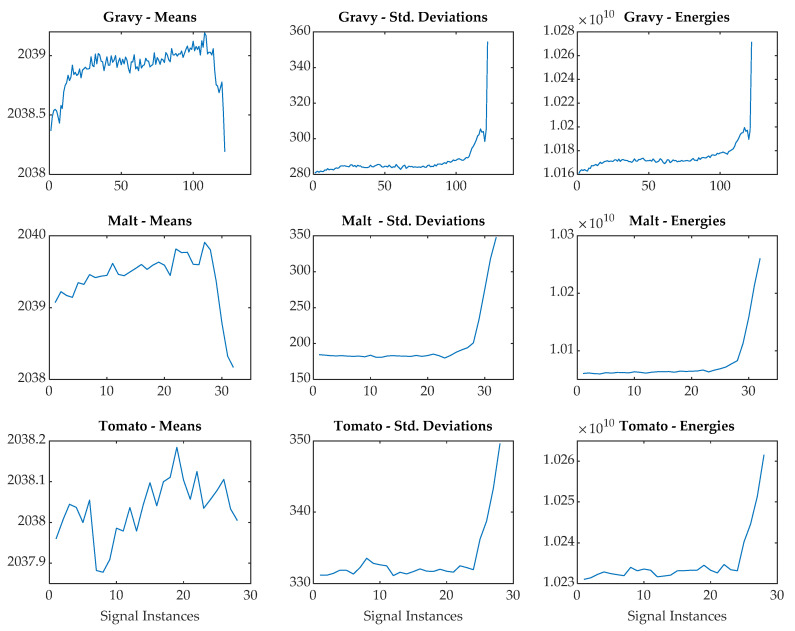
Signal features examples for the gravy, malt, and tomato.

From the decomposition vector, the approximation coefficients were computed for all the signal instances. Subsequently, from the approximation coefficients, a feature extraction procedure was applied to compute a number of statistical features [43], namely mean, standard deviation, minimum, maximum, skewness, kurtosis, and energy. An example of the most significant features is reported for the various fouling materials in Figure 6 with the aim of showing the trends during the cleaning process.

The results in Figure 6 displays how three different statistical features (means, standard deviations, and energies) change during cleaning for the three different fouling materials. For all materials, the energies and standard deviations follow similar trends with an increase toward the end of the process when the test section becomes clean. Although the starting values for the standard deviations and energies are different for the three fouling materials, they all reach a similar end point once clean, which was approximately 350 for the standard deviations and 1.03 × 10^10^ for the energies. The results for the means showed a different trend of gradually increasing during the initial stage of the cleaning process before suddenly reducing at the end. The means for the tomato did not show this trend, indicating that one statistical method may not be appropriate for monitoring the processes. It is not surprising to see that the largest change in the US statistical features was only at the end of the process, as previous work has shown that US reflection methods are only sensitive to the area of fouling covering the area on the test section opposite the transducer location and not the thickness of this fouling.

#### 2.2.2. Image Processing

The quantification of the surface fouling and fouling volume required for the training dataset was carried out via image processing on the images acquired during the cleaning process.

**Figure 7:**
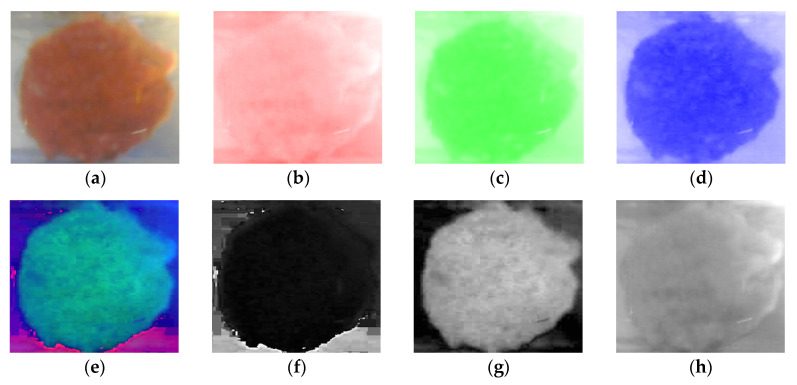
Red–Green–Blue (RGB) image (**a**), red channel (**b**), green channel (**c**), and blue channel (**d**), Hue–Saturation–Value (HSV) image (**e**), hue channel (**f**), saturation channel (**g**) and value channel (**h**).

The idea is to manipulate the images to highlight relevant information about the fouling. An initial pre-processing step for all the digital images is the channel separation. In this respect, the raw image in Figure 7a was acquired in Red–Green–Blue (RGB) space, consisting of three different layers, i.e., the red, green, and blue, separately shown in Figure 7b–d.

The raw RGB image was subject to a transformation procedure to be mapped into Hue–Saturation–Value (HSV) values [44]. The image channel breakdown is reported in Figure 7 with reference to a Tomato Cold 1 image instance. The HSV domain (Figure 7e) is characterized by three layers, corresponding respectively to the Hue (Figure 7f), i.e., the colour’s position on the colour wheel, the Saturation (Figure 7g), i.e., amount of hue or departure from neutral (zero denotes a neutral shade, whereas 1 indicates maximum saturation), and Value (Figure 7h, i.e., the maximum value among the red, green, and blue components of a specific colour). 

Taking into account the experimental conditions, in terms of light source, food fouling colours, and camera settings, a tailored image processing procedure was developed in this research for quantifying the fouling volume. From the separated channels, a new image I is computed as reported in Equation (2).

**Figure 8:**
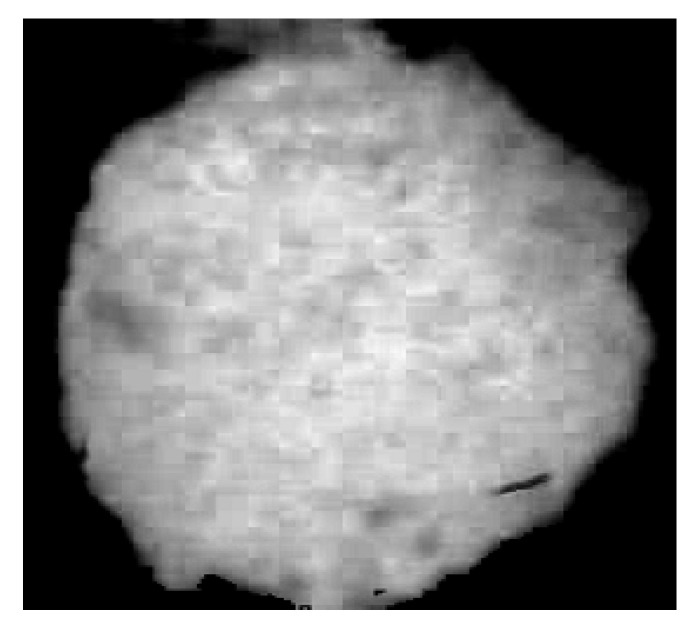
Processed image for fouling estimation.

(2) I=(∁(R−S))−B−H

where R is the red channel, S is the saturation channel, B is the blue channel, H is the hue channel, and the operator ∁ indicates the image complement (negative image). In such an image, the background results are removed, and the pixel intensity results to be proportional to the fouling volume, as it can be seen in the result shown in Figure 8.

**Figure 9:**
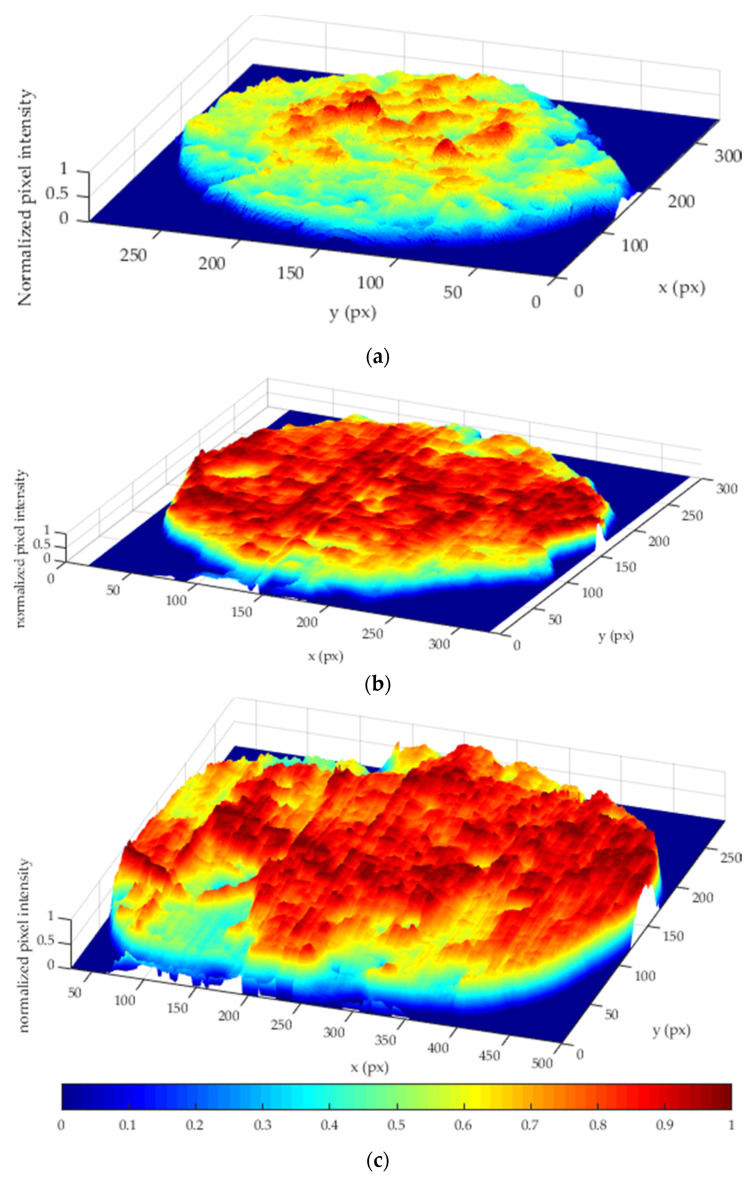
3D fouling volume representation. (**a**) Gravy, (**b**) tomato, and (**c**) malt.

The fouling volume can be graphically visualized as the pixel intensity (*z*-axis) as a function of the image pixels (*x* and *y* axes) shown in the 3D plot reported in Figure 9, where the pixel intensity was normalized between 0 and 1 for an enhanced visualisation.

Then, the computation of the fouling volume for the single image instance is computed as the sum of the pixel intensities, i, within the image *I*, as reported in Equation (3).

(3) $$\mathrm{Fouling}\;\mathrm{Volume}=\overset{m\times n}{\underset{p=1}{\sum }}i_{p}$$

where m and n are the vertical and the horizontal resolution of the image.

In order to estimate the amount of fouling, a Fuzzy c-means clustering-based thresholding method [34] was applied to the processed image following the steps listed below: 

The FCM is carried out by minimizing a certain objective function as shown in Equation (4)

(4) $$J_{m}=\overset{N}{\underset{i=1}{\sum }}\overset{C}{\underset{j=1}{\sum }}\mu_{ij}^{m}||x_{i}-c_{j}||{}^{2}$$

where *N* is the number of the data points, here represented by the image pixels ($x_{i}$), C is the number of clusters, and m is the fuzzy partition exponent corresponding to the fuzzy overlap degree. $c_{j}$ is the center of the $j^{th}$ cluster and $\mu_{ij}$ is the degree of membership of each pixel ($x_{i}$) in the $j^{th}$ cluster. For the image segmentation proposed in this paper, the steps for the FCM implementation are as follows:

- Set the number of clusters C=3, respectively “small”, “medium”, and “large” based on the pixel intensity value. The fuzzy matrix exponent m was set to 2.
- Initialize the cluster membership with random $\mu_{ij}$ values.
- Compute the cluster centers according to Equation (5):
  (5) $$c_{ij}\frac{\sum_{i=1}^{N}\mu_{ij}^{m}x_{i}}{\sum_{i=1}^{N}\mu_{ij}^{m}}$$
- Update $\mu_{ij}$ as per Equation (6):
  (6) $$\mu_{ij}=\frac{1}{\sum_{k=1}^{C}{\left(\frac{\left||x_{i}-c_{j}|\right|}{\left||x_{i}-c_{k}|\right|}\right)}^{\frac{2}{m-1}}}$$
- Compute the value of the objective function $J_{m}$
- Recompute $c_{ij}$, $\mu_{ij}$, and $J_{m}$ until meeting a termination criterion, such as a minimum improvement or maximum number of iterations.
- Retrieve the final centroids coordinates and the final fuzzy membership degree of each piece of pixel data.
- Assign each pixel to one of the three clusters based on the maximum membership degree.
- Compute the maximum pixel intensity value for the cluster “small” $\max\left(i_{small}\right)$ and the minimum pixel intensity value for the cluster “medium” $\min\left(i_{medium}\right)$.
- Compute the threshold value as the average of the two values computed above, i.e.,
  (7) $$T=\frac{\left[\max\left(i_{small}\right)-\min\left(i_{medium}\right)\right]}{2}.$$

**Figure 10:**
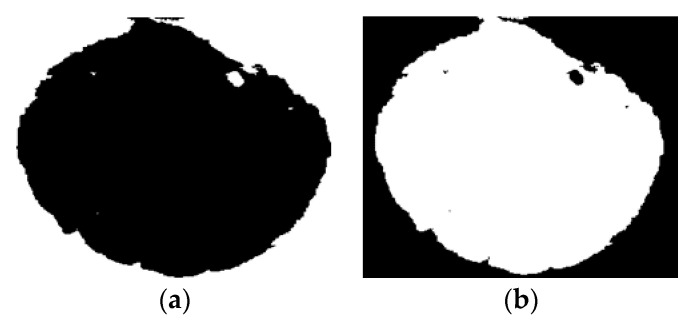
Image segmentation steps: Fuzzy c-means (FCM) segmented image (**a**), negative (**b**).

An example of segmented image using the FCM thresholding technique is reported in Figure 10a. To facilitate the computation process, the segmented image was then transformed to its negative, as shown in Figure 10b.

From the negative segmented image, the surface fouling is computed as the sum of the pixel intensities, $i_{p}$, as reported in Equation (8), where m and n are the horizontal and vertical image resolution, respectively.

(8) $$\mathrm{Surface}\;\mathrm{Fouling}\;=\overset{m\times n}{\underset{p=1}{\sum }}i_{p}$$

**Figure 11:**
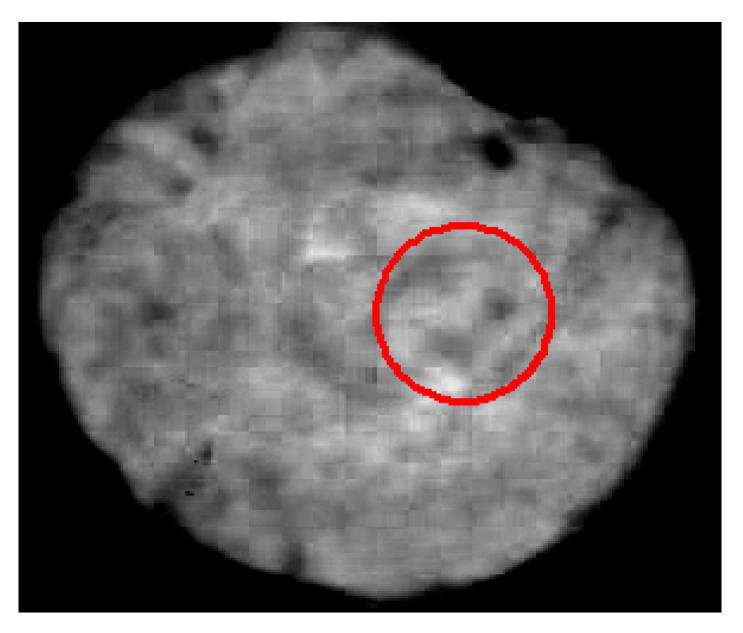
Region of Interest (ROI).

Within the acquired image, a Region of Interest (ROI) was determined in correspondence of the sensor positioning; therefore, the surface fouling and the fouling volume were computed only for the ROI area, i.e., a circle with radius = 45 pixels, as depicted in Figure 11.

**Figure 12:**
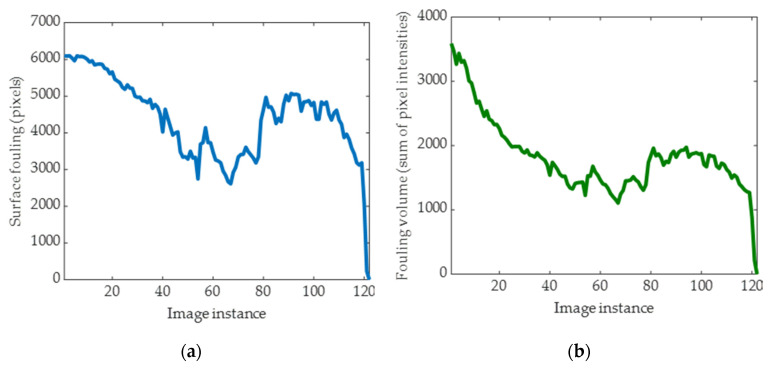

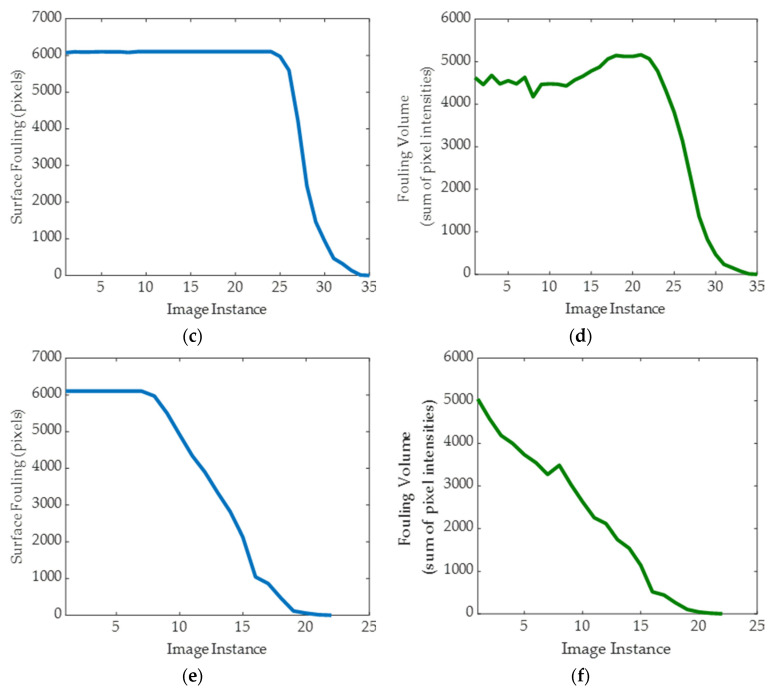
Surface fouling for gravy_cold_1 (**a**), fouling volume for gravy_cold_1 (**b**), surface fouling for malt_cold_2 (**c**), fouling volume for malt_cold_2 (**d**), surface fouling for tomato_cold_1 (**e**), and fouling volume for tomato_cold_1 (**f**).

Figure 12 shows examples of surface fouling and fouling volume trends. The increasing trend occurring in some of the samples is explained by the fact that during the cleaning process, fouling material lumps are progressively smeared over a larger surface [14].

As regards fouling volume charts, the fluctuations are due to the small size of the ROI and the transient nature of the fouling, i.e., during the cleaning process, fouling may temporarily increase in the ROI as it is moves across the surfaces [14,16].

The results in Figure 12 show that for all three fouling materials, the number of fouled pixels and the fouling volume reduces in the ROI during the cleaning processes and achieves a value of zero when all fouling has been removed.

Although the general trend of reduction is consistent between the surface fouling and fouling volume for the different fouling materials, differences do exist within the results. These are most noticeable for tomato where fouling volume (Figure 12f) begins to reduce at the start of the cleaning process, whereas surface fouling (Figure 12e) does not begin to reduce until after approximately the seventh image instance. This result indicates that volume fouling is a more useful technique to monitor cleaning processes; however, this trend was not identified with the gravy or malt (Figure 12a–d). For the gravy, both the surface fouling and fouling volume followed a similar trend of reducing until the 80th image instance before increasing slightly. The volume fouling for the malt also appeared to increase marginally between the image instances of 15 and 25. Although it would not be expected for the amount of fouling to increase during cleaning processes, there are numerous explanations for this result. Only a small ROI of interest is been analysed, and it is possible that fouling from the surrounding area has moved into this ROI due to the mechanical motion of the flowing fluid. For the case of the gravy, it is known that this swells as it absorbs water and also becomes partly detached from the base of the surface [27], resulting in a lump of gravy that would move slightly closer to the top of the rig and nearer the camera, resulting in more fouling within the ROI. It was not surprising to identify different results for surface fouling and fouling volume between the three fouling materials studied. It is well known that materials with different physicochemical properties foul and are removed from surfaces differently [37]. Previous research has shown that tomato is removed primarily by mechanical force, whereas gravy initially swells due to moisture absorption before been removed as a bulk lump, and malt dissolves gradually into the fluid [27]. 

### 2.3. Machine Learning Regression for Surface Fouling and Fouling Volume Estimation 

The estimation of surface fouling and fouling volume was modelled in this research as a regression problem from an ultrasonic signal. In this respect, the statistical features from wavelet decomposition vector (partially illustrated in Figure 6) were inputted to a Neural Network data fitting [25] decision-making support system for surface fouling quantification purposes. Three-layer feed-forward neural networks were built with the following architecture:

- Input layer nodes corresponding to the seven features extracted from the wavelet approximation coefficients
- Hidden layer nodes (HLN): 7
- Target layer: one node corresponding to the number of white pixels computed via image processing
- The training algorithm adopted in this research was the Bayesian Regularization (BR) [27].
- The dataset was partitioned into three sets using specified indices, specifically alternating one instance for training, one for validation, and one for testing [22].

## 3. Results and Discussion

Regression results are characterized by two indicators of the goodness of fitting, namely the Root Mean Squared Error (*RMSE)* and the correlation coefficient *R*, which are computed according to Equations (9) and (10).

(9) $$RMSE=\sqrt{\frac{1}{N}\overset{N}{\underset{i=1}{\sum }}{\left(y_{i}-{\hat{y}}_{i}\right)}^{2}}$$

(10) $$R=\sqrt{1-\frac{\sum_{i=1}^{N}{\left(y_{i}-{\hat{y}}_{i}\right)}^{2}}{\sum_{i=1}^{N}{\left(y_{i}-\bar{y}\right)}^{2}}}$$

where, with reference to both surface fouling and fouling volume, $y_{i}$ are the actual values, ${\hat{y}}_{i}$ are the values predicted by the NN, and $\bar{y}$ represents the average values.

The results for all the tests are reported in Table 2 and Table 3, both in terms of *R* coefficient and *RMSE* for all the tests, which are divided in categories.

Results are shown for the three different NN phases. In this respect, the training results correspond to the NN performance obtained by using the training samples including the validation samples, accounting for two-thirds of the whole dataset. The test results correspond to the remaining one-third of the dataset samples used to train the system. The overall results show a weighted average of the training and test results.

The regression results for surface fouling (Table 2) and fouling volume (Table 3) show acceptable results with *R* coefficient values above 0.8 for all models developed and above 0.9 for many. There was no clear difference in regression accuracy between the surface fouling and fouling volume models or in the models developed for the different fouling materials. 

In general, the models developed for the cleaning experiments at the lower temperature had better model performance, but this is most likely because the fouling took a longer time to clean at the lower temperature, so more data were available to train the models. 

It is difficult to directly compare these results with those from the literature, as the authors are not aware of previous research that has combined US measurements with neural networks to develop regression models that are capable of monitoring cleaning processes. However, previous work has used similar methods to monitor mixing progress with similar *R* values reported [45].

The model performance could be improved with more training data, which could be achieved by either (1) performing more experiments, (2) increasing the frequency of data collection during the experiments, or (3) adding additional optical and ultrasonic sensors to the experimental rig. From an industrial perspective, large amounts of data would be collected during routine CIP cycles, allowing the development of highly accurate and reliable regression models. Indeed, for industrial implementation, datasets would need to be generated for each installation and fouling type to develop accurate monitoring capabilities, thus improvements in performance are inherent with application.

**Figure 13:**
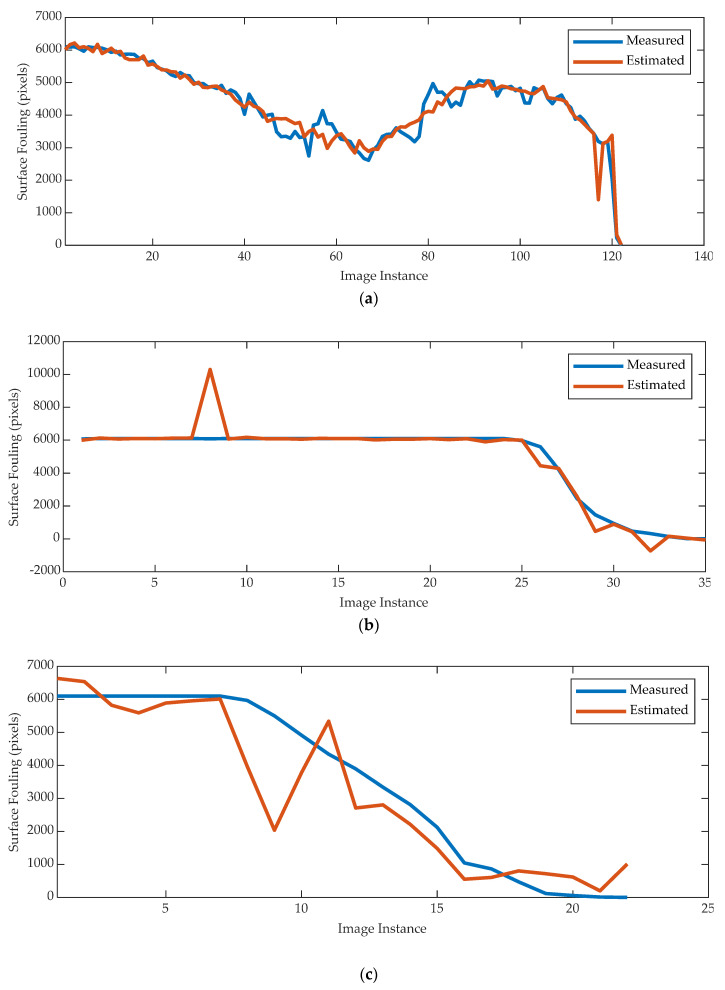
Measured surface fouling versus NN estimations for gravy_cold_1 (**a**), malt_cold_2 (**b**), and tomato_cold_1 (**c**) tests.

**Figure 14:**
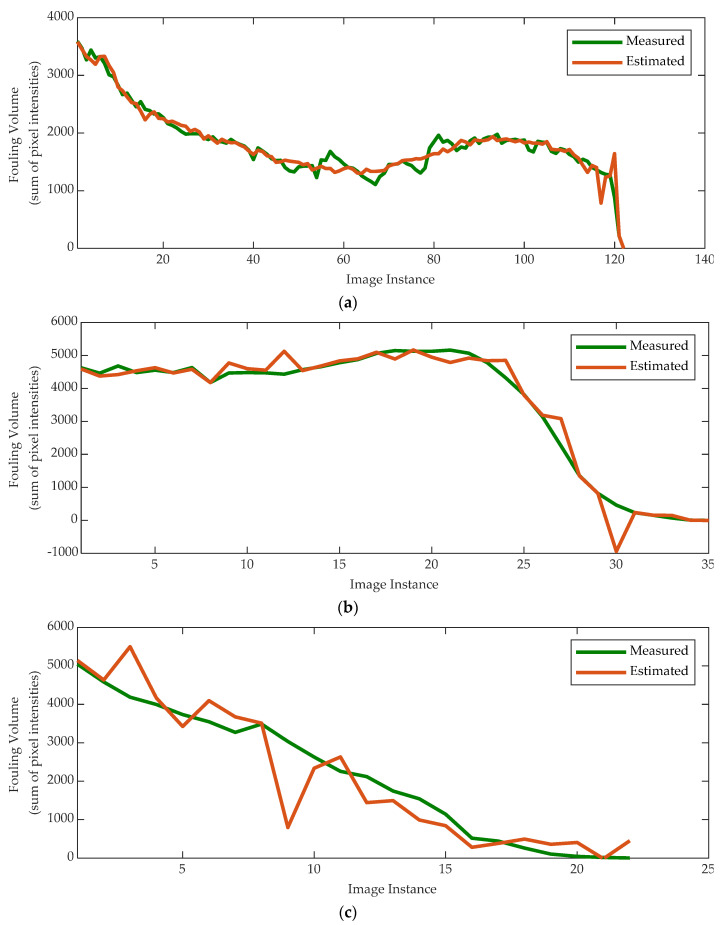
Measured fouling volume vs. NN estimations for gravy_cold_1 (**a**), malt_cold_2 (**b**), and tomato_cold_1 (**c**) tests.

Figure 13 and Figure 14 display the predicted and measured surface fouling and fouling volume for the three different materials. In general, the predicted results are in good agreement and follow the trend of the measured results, highlighting the potential of the technique. There appears to be less error between the predicted and measured results for the fouling volume, whilst the predictions of tomato fouling have the most error, but this could be attributed to the fact that there was less data available to train the models. 

The analysis of the charts in Figure 13 and Figure 14 highlights how different materials yield to different cleaning trends and different NN performance in terms of regression accuracy.

The main factor explaining the presence of such outliers is represented by the number of available samples. Meanwhile, the gravy absorbs the water and requires longer time to perform the cleaning process, resulting in a larger amount of data, therefore yielding to a more accurate regression. In contrast, the malt and tomato require a shorter cleaning time as they instead dissolve. Consequently, the number of available samples results is smaller thus; although overall accurate, the regression shows a (small) number of outliers.

From a strictly computational point of view, this can be further explained by how the nature of the fouling material affects the cleaning trend. As regards the malt and tomato, the surface fouling plots show a cleaning trend characterized by a two-phase process, i.e., a “flat” one and a “rapid descent” one. This means that the neural network is trained and tested with a high rate of different input signals but very similar target values, yielding to a lower regression performance.

## 4. Conclusions

This work has demonstrated how ultrasonic and optical sensors can be used to monitor surface fouling removal and therefore cleaning processes. Three different food fouling materials (tomato, gravy, and malt) were studied, and it was found that although they all cleaned via different mechanisms, they could all effectively be monitored via the sensing methods. The optical sensor provided spatial information on the area and volume of fouling in the region of interest, providing a greater insight to the fouling removal process than the ultrasonic method. However, it would be extremely difficult to effectively image the fouling in industrial equipment such as pipe work, so the ultrasonic method would be a more suitable industrial technology. Both sensors could only provide information on a small area of equipment, so care must be taken when deploying this in industrial environments to determine the most effective number of sensors to utilize and the precise locations to place these sensors. A larger number of sensors would provide more data but would come with additional costs. The regression machine learning models were effective at predicting the area and volume of fouling present from the ultrasonic measurements. In this respect, the surface fouling was estimated with an average *RMSE* of 746.265 (from 156.967 to 1157.345) and an average *R* of 0.921 (from 0.832 to 0.9978), while the fouling volume was estimated with an average *RMSE* of 650.498 (from 112.961 to 1374.544) and an average *R* of 0.8927 (from 0.708 to 0.983). A detailed experimental validation would be required to establish a quantitative correspondence between pixel intensity and fouling thickness under the adopted experimental conditions, i.e., lights and camera, which were finalized upon determining the maximum thickness detectability. In industry, the accuracy of prediction would be improved with the collection of larger datasets used to train the models, which would be generated for each installation and fouling material.

Additionally, outliers and missing data have been manually removed in a preliminary data screening phase. In this respect, future work will also include an automatic data debugging procedure that can be applied to detect evident outliers and image artefacts that could affect the processing and NN performance, yielding to misleading results.

The experimental limitations of the proposed monitoring systems are certainly represented by the number and the small size of the region of interest ROI, i.e., a single ROI of roughly 6000 pixels, as well as by the limited range of water temperature used cleaning, i.e., 12 °C and 45 °C and by the limited number of different fouling materials. Other limitations include the use of only a single point measurement, which may not be sensitive to fouling in the rest of the equipment. 

A limitation to the industrial implementation is represented by operational difficulty in the proper positioning of the US sensors within an existing industrial equipment, especially in the presence of heater jackets on the pipes.

Future research is aimed at determining the exact number of sensors as well their optimized positioning. Additionally, the cleaning performance of different types of fouling materials characterized by various concentrations of fats, minerals, and proteins has to be investigated. Finally, efforts will be focused on integrating the sensing units with a control system. Similar issues can be encountered in the image acquisition process, which is required to label the US data and needs to be carefully designed to be implemented in existing facilities.

## Figures and Tables

**Table 1:** Experimental Programme.

Fouling Material	Temperature	Repetitions
Gravy	12 °C	2
	45 °C	7
Malt	12 °C	7
	45 °C	7
Tomato	12 °C	7
	45 °C	7

**Table 2:** Summary of surface fouling Neural Network (NN) regression results. RMSE: Root Mean Squared Error.

Surface Fouling						
Dataset	*R*			*RMSE*		
	Training	Test	Overall	Training	Test	Overall
Gravy Cold	0.9210	0.8923	0.9175	360.4690	363.6912	361.6612
Gravy Hot	0.9275	0.9355	0.9285	723.6536	660.2220	700.1839
Gravy Cold+Hot	0.9271	0.8486	0.9149	544.6095	790.3567	635.5366
Malt Cold	0.9978	0.9132	0.9635	156.9672	1142.7719	521.7149
Malt Hot	0.9462	0.8524	0.9195	744.4553	1131.9732	887.8369
Malt Cold+Hot	0.9813	0.9595	0.9784	469.5991	656.6640	538.8131
Tomato Cold	0.9439	0.9056	0.9305	670.1577	914.743	760.6543
Tomato Hot	0.9359	0.9256	0.9324	832.4331	883.7525	851.4213
Tomato Cold+Hot	0.9104	0.9136	0.9075	912.20	1023.70	953.4550
COLD	0.9395	0.9310	0.9366	616.4055	659.4567	632.3344
HOT	0.9238	0.8576	0.9006	838.0589	1157.3449	956.1947
ALL	0.9173	0.8320	0.8877	783.6593	1120.2075	908.1821

**Table 3:** Summary of fouling volume NN regression results.

Fouling Volume						
Dataset	*R*			*RMSE*		
	Training	Test	Overall	Training	Test	Overall
Gravy Cold	0.9673	0.9307	0.9337	112.9609	130.3614	119.3991
Gravy Hot	0.8169	0.8025	0.8119	800.9874	857.0363	821.7255
Gravy Cold+Hot	0.9119	0.7079	0.8772	466.5958	869.9793	615.8477
Malt Cold	0.9728	0.7890	0.9027	453.6082	1374.5441	794.3545
Malt Hot	0.9461	0.8024	0.9016	669.7703	1174.9157	856.6741
Malt Cold+Hot	0.9336	0.9121	0.9301	734.3514	815.3636	764.3259
Tomato Cold	0.9498	0.8422	0.9116	413.8871	739.6857	534.4326
Tomato Hot	0.9833	0.9399	0.9690	289.2188	546.4321	384.3877
Tomato Cold+Hot	0.9232	0.9071	0.9176	573.7849	623.9646	592.3514
COLD	0.9387	0.9095	0.9287	493.7523	603.0507	534.1927
HOT	0.8584	0.8201	0.8453	789.2190	901.1648	830.6389
ALL	0.9243	0.8264	0.8897	571.2305	878.7315	685.0059
